# Forward Genetic Analysis of Visual Behavior in Zebrafish

**DOI:** 10.1371/journal.pgen.0010066

**Published:** 2005-11-25

**Authors:** Akira Muto, Michael B Orger, Ann M Wehman, Matthew C Smear, Jeremy N Kay, Patrick S Page-McCaw, Ethan Gahtan, Tong Xiao, Linda M Nevin, Nathan J Gosse, Wendy Staub, Karin Finger-Baier, Herwig Baier

**Affiliations:** Department of Physiology, Programs in Neuroscience, Genetics, and Developmental Biology, Center for Human Genetics, University of California, San Francisco, California, United States of America; University of Pennsylvania School of Medicine, United States of America

## Abstract

The visual system converts the distribution and wavelengths of photons entering the eye into patterns of neuronal activity, which then drive motor and endocrine behavioral responses. The gene products important for visual processing by a living and behaving vertebrate animal have not been identified in an unbiased fashion. Likewise, the genes that affect development of the nervous system to shape visual function later in life are largely unknown. Here we have set out to close this gap in our understanding by using a forward genetic approach in zebrafish. Moving stimuli evoke two innate reflexes in zebrafish larvae, the optomotor and the optokinetic response, providing two rapid and quantitative tests to assess visual function in wild-type (WT) and mutant animals. These behavioral assays were used in a high-throughput screen, encompassing over half a million fish. In almost 2,000 F2 families mutagenized with ethylnitrosourea, we discovered 53 recessive mutations in 41 genes. These new mutations have generated a broad spectrum of phenotypes, which vary in specificity and severity, but can be placed into only a handful of classes. Developmental phenotypes include complete absence or abnormal morphogenesis of photoreceptors, and deficits in ganglion cell differentiation or axon targeting. Other mutations evidently leave neuronal circuits intact, but disrupt phototransduction, light adaptation, or behavior-specific responses. Almost all of the mutants are morphologically indistinguishable from WT, and many survive to adulthood. Genetic linkage mapping and initial molecular analyses show that our approach was effective in identifying genes with functions specific to the visual system. This collection of zebrafish behavioral mutants provides a novel resource for the study of normal vision and its genetic disorders.

## Synopsis

While many genes have previously been implicated in the development and function of the vertebrate central nervous system, no systematic attempt has been made to build a comprehensive catalog of genes important for its behavioral output. Motion evokes two visual reflexes in zebrafish larvae, the optomotor and the optokinetic response. After mutagenesis with ethylnitrosourea and inbreeding over two generations, the authors of this study searched for point mutations disrupting either, or both, of these innate responses. In almost 2,000 F2 families, they discovered 53 recessive mutations in 41 genetic loci. Developmental phenotypes included abnormal differentiation or absence of photoreceptors, and deficits in retinal ganglion cell differentiation or axon targeting. Physiological phenotypes include disruptions of phototransduction, light adaptation, and behavior-specific responses. Most of the mutants are morphologically indistinguishable from wild type, and many survive to adulthood. Genetic linkage mapping and initial molecular analyses revealed that the authors' approach identified genes with functions specific to the visual system. This collection of zebrafish behavioral mutants provides a novel resource for studying the genetic architecture of the vertebrate central nervous system.

## Introduction

An animal's behavioral repertoire is deeply rooted in its genome. Mutations of behaviorally important genes may alter or disrupt either the physiology of neuronal circuits or their development. The first task of a research program aimed at identifying the genetic underpinnings of perception and behavior is to build a comprehensive catalog of genes with specific, non-lethal phenotypes, initially with no regard of when and where in the organism they are acting. Forward genetic screens are the method of choice to identify those genes in an unbiased fashion. This approach was pioneered over 30 years ago by Benzer in *Drosophila melanogaster* [1] and was quickly extended to *Caenorhabditis elegans* [2]. In these invertebrate species, the forward genetic strategy was particularly productive for the analysis of sensory systems, such as vision, mechanosensation, and olfaction, where these screens helped to discover many genes important for the patterning of sensory epithelia and for sensory transduction [3–7].

Very few behavioral screens have been attempted in vertebrates to date. In mice, Takahashi and colleagues carried out a screen for dominant mutations disrupting circadian behavior [8]. Other groups have carried out behavioral “shelf screens” of previously discovered mutants in both zebrafish and mice [9–11] or collected mutants in motility and locomotor coordination [12,13]. Here we report on the results of the first large-scale behavioral screen focused on a vertebrate sensory system. Following chemical mutagenesis, we searched for recessive mutations that disrupt visually evoked behaviors in zebrafish*.* Brockerhoff et al. first showed the utility of optokinetic behavior as a powerful screening tool to find visual mutants [14]. Here we used both the optokinetic response (OKR) and the optomotor response (OMR) as screening assays [9,14–16]. These two behaviors employ different motor outputs (swimming and eye movements, respectively), but they are both elicited by large-field motion and are dependent on the retina as the light-sensing organ [15,17]. In a high-throughput screen of almost 2,000 mutagenized genomes, we discovered 41 loci whose mutations lead to a broad spectrum of specific visual (or visuomotor) impairments. Some of the more striking phenotypes include new mutants in retinal axon targeting and in the adaptive dynamics of light responses. This first survey reveals the extent to which single-gene mutations can perturb visual behavior without affecting gross development or vital organ functions. The identities of the corresponding genes are beginning to provide novel insights into how the visual system is assembled and how cellular and molecular interactions shape sensory processing in the vertebrate brain.

## Results

### Design of an Efficient, Large-Scale Mutagenesis Screen in Zebrafish

We carried out a large-scale screen for mutants with defects in visually elicited behavior. Forty-one founder males (F0) treated with ethylnitrosourea (ENU; see Materials and Methods) were mated with wild-type (WT) females to generate more than 5,000 F1 fish. Adult F1 fish were mated with other F1 fish, or with WT fish, to generate more than 2,000 F2 families. In total, 3,171 F1 fish were used to generate the 1,896 F2 families (2,550 F1 fish for F1 × F1 crosses, and 621 F1 fish for F1 × WT) that gave at least one healthy clutch of F3 embryos in the subsequent generation. F3 embryos and larvae were obtained by random crosses between siblings from F2 families (6,468 F3 clutches in total, or 3.4 clutches per each F2 family on average). From each F3 clutch, typically 12 larvae were tested for OKR and 25 larvae for the OMR (see below). Fish were routinely scored on the seventh day postfertilization (7 dpf). Including retests, over 500,000 individual fish were screened in the course of three years. Calculations based on binomial statistics [18], taking into account the number of F1 fish used to generate the F2 families, the number of F2 families, the number of crosses for each F2 family, and the number of F3 larval fish tested, show that our screen encompassed 1,688 ENU-mutagenized genomes.

The efficiency of mutagenesis in the founder male germlines was determined by a specific-locus test, using *sandy (sdy),* a zebrafish tyrosinase mutant [19]. In this test, ENU-treated founder males mated with *sdy* heterozygous females produced six new *sdy* mutations in about 2,000 genomes screened. In the actual screen of F2 families, however, two new *sdy* mutant alleles were identified. The allele distribution of all loci, which was determined after completion of the screen and following extensive complementation tests, shows that our screen was not saturated (see Discussion). We nevertheless successfully identified new alleles of previously reported visual mutants, such as *bel* and *nof* (Table 1). Although we did not attempt to characterize mutations falling outside our screening criteria, i.e., those causing embryonic or larval lethality, we noticed (and most of the time discarded) new alleles of *chk* [20], *bru/eby* [21,22], *ome,* and *nok* [21] (unpublished data).

**Table 1:**
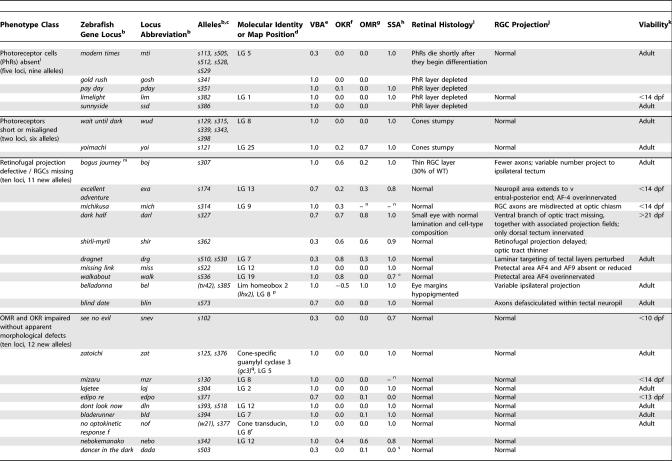
Continued

**Table 1:**
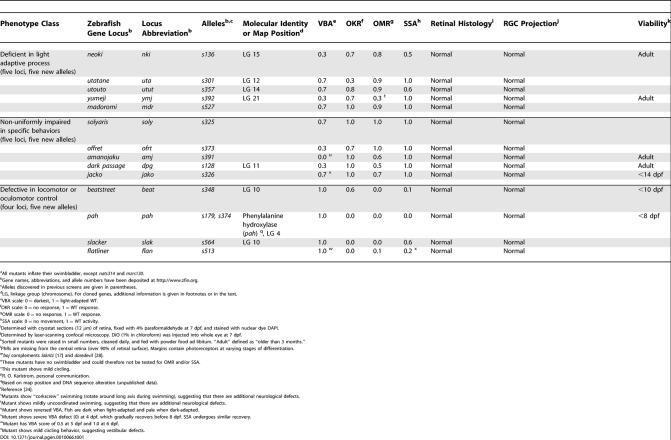
Zebrafish Visual Behavior Mutants^a^

### Two Behavioral Screening Assays, Executed in Parallel, Discovered 53 Visual Mutants

**Figure 1:**
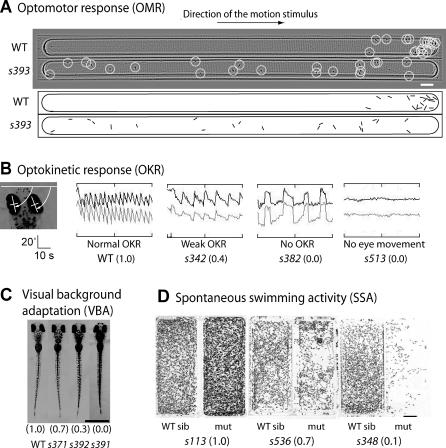
Behavioral Screening Assays (A) OMR. WT larvae in the racetrack reflexively swim in the same direction as a moving stimulus (top). Mutant larvae (for example, *dln^s393^*) with an OMR index of 0 fail to respond (bottom). A contrast-enhanced image outlining the fish is shown in the lower image. In this experiment, WT fish larvae were driven all the way to the right end of the racetrack, which differs slightly from our screening assay [16]. (B) OKR. Eye positions (angles shown by white arrows, far left image) were plotted over time during optokinetic stimulation in one direction. The OKR has a sawtooth profile, consisting of alternating quick and slow phases. OKR mutants show slowed eye movements (for example, *nebo^s342^*), absence of the OKR *(lim^s382^),* or no eye movements *(flan^s513^)*. Corresponding OKR indices are given in parentheses. (C) VBA. WT (VBA index = 1) shows fully contracted melanophores in bright illumination. Mutants *(edpo^s371^, ymj^s392^,* and *amj^s391^)* show three gradations of darker pigmentation, due to enhanced melanin dispersal. Scale bar is 1 mm. (D) SSA. Movies of six fish per rectangular well, taken at 0.5 frame per second for 20 min, were subtracted frame by frame and projected into a single image to show the locomotor behavior over time. Blind mutants, such as *mti^s113^* (OKR and OMR indices = 0), may show normal spontaneous activity (SSA index = 1). The *mti* mutants are also darker (VBA = 0.3), resulting in a higher-contrast image than WT. The *walk^s536^* mutants (OKR = 0.8; OMR = 0) show less activity, with some circling (SSA = 0.7), which could explain part of their OMR defect. In *beat^s348^* mutants, locomotion is severely compromised (SSA = 0.1). SSA-defective mutants were not systematically kept.

We screened for mutations disrupting behavioral responses to visual motion. A coarse grating that drifts across the fishes' visual field elicits either of two distinct responses, an OKR or an OMR. In the OMR, WT animals vigorously swim in the direction of the perceived motion (Video S1). When restrained from swimming and presented with a rotating whole-field motion stimulus, the fish show an OKR to cancel retinal slip: WT animals move their eyes to track the motion. These pursuit phases are interrupted at regular intervals by reset movements, or saccades (Video S2) [15]. To achieve high throughput, we automated both visual stimulation and analysis, as described elsewhere [16]. We found that the two screening assays were complementary: The OKR assay is slower and more labor-intensive, but has single-fish resolution; the OMR assay, on the other hand, is fast, but measures only population responses. For each assay, a behavioral index ranging from 0 (no response) to 1.0 (WT) was calculated (see Materials and Methods). Typical OMR and OKR mutant phenotypes are shown in Figure 1A and 1B.

Mutants detected by at least one of the two assays in the primary screen were kept. To select against phenotypes with general defects, we discarded mutants with overt developmental problems, as well as those that were poor swimmers, with a few exceptions. Putative F2 carriers were mated at least twice more for confirmation of the phenotype in their progeny before they were outcrossed. The OKR screen initially picked up 241 putative mutants, or “putants.” Following two retests, 46 lines (23%) were outcrossed. The OMR screen picked up 361 putants, 34 (9%) of which were confirmed and successfully propagated. In addition to high-contrast stimuli, we also routinely used a lower-contrast grating to detect subtle and/or contrast-specific visual defects. The high percentage of false positives is mostly attributable to the use of these weak test stimuli. The OKR and OMR assays were used independently within the primary screen. A considerable number of OKR mutants were later found to be OMR-deficient, and vice versa, as discussed below (Table 1).

The initial false positive rate of this behavioral screen greatly exceeded that of a morphological screen for small-eye mutants carried out in parallel [23]. However, almost all behavioral mutants were recovered in the following generation. Our strategy of extensive retesting as part of the primary screen therefore dramatically decreased the number of false positives and made this screen practical. Mutants or putative mutants with low penetrance were not kept or are not reported here. The mutants presented in this paper, therefore, were found in about 25% of the population in a clutch. To establish potential complementation groups, we systematically crossed heterozygous carriers of mutants with similar phenotypes. Noncomplementing mutations (in which the transheterozygous progeny showed a mutant phenotype) were considered to be allelic (Table 1).

### Secondary Screening Assays Allowed Classification of Behavioral Phenotypes

In addition to OMR and OKR, we also assessed the larvae's visually mediated background adaptation (VBA) at 5 dpf, as a complementary strategy to enrich for visual mutants. The VBA is a neuroendocrine response that is controlled by ambient light levels and appears to depend on the function of retinal ganglion cells (RGCs) [17]. Melanophores in the skin contract their melanin granules in a bright environment, while a dark environment induces melanin dispersal [9]. We tested the VBA only in response to long (over 20 min) exposure to bright light, i.e., the mutants' ability to become pale. Figure 1C shows gradations of the VBA defect in three representative mutants. We found that, of the 89 VBA mutants discovered in the screen, 19 (21%) also had specific OMR or OKR defects. The remaining 70 “dark” mutants were either behaviorally normal or had externally visible, morphological phenotypes and were not always maintained.

To identify defects in motor functions, we systematically tested spontaneous swimming activity (SSA) (Figure 1D) in all our mutants. We also made sure that all mutants listed in Table 1, except *s513,* showed spontaneous, conjugate eye movements similar to WT when presented with a stationary stimulus. Finally, to identify mutants with developmental defects, we systematically examined their retinal and tectal histology and their retinotectal projections (Table 1).

### Mutations May Affect Some Visual Behaviors More than Others

**Figure 2:**
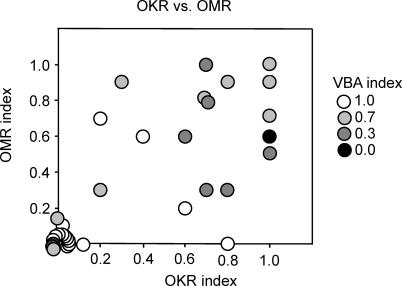
Distribution of Behavioral Phenotypes among the Three Visual Responses OMR index is plotted over OKR index for each mutant. Each circle represents a mutant. The shading of the circles represents the VBA index for that mutant. Only mutants with SSA index greater than 0.6 are shown. OMR is strongly correlated with OKR only for very low scores (around 0). Mildly impaired mutants are often differentially affected. OMR and OKR performance is not correlated to VBA index.

Because OKR and OMR are both evoked by motion of a large field grating, but differ in their motor output, our collection of mutants presented us with an opportunity to ask how well single-gene mutations can dissociate these two related behaviors. Are there mutations that impair OMR and OKR in a differential manner (weak dissociation) or even disrupt only one of the behaviors, while leaving the other unaffected (strong dissociation)? Table 1 shows that none of our mutants showed a complete absence of either OMR or OKR together with no defect at all in the other behavior. However, the two behaviors were often affected to different degrees. To reveal potential correlations, we plotted the behavioral profiles of our mutant set (Figure 2). Each data point in Figure 2 corresponds to one mutant, measured repeatedly (*n* > 3 clutches), and was also shaded to represent that mutant's light-exposed VBA score. Although many mutants lacked any visual responses, for those with partial OMR and OKR phenotypes, there was no clear relationship between the magnitudes of the deficit in the two behaviors (correlation coefficient *r* = 0.4, when mutants with OKR = 0 and OMR = 0 were excluded). Perhaps surprisingly, the severity of the VBA phenotype was not positively related to either OMR (*r* = −0.5) or OKR (*r* = −0.4) defects. The overall correlation of all OMR and OKR indices (*r* = 0.75) and the absence of exclusively OMR- or OKR-specific mutants suggest that these behaviors are weakly dissociable by single-gene mutation. This indicates that OMR and OKR share a major portion of the underlying neural circuitry. In contrast, the VBA appears to employ a dedicated neural pathway largely segregated, and therefore genetically separable, from motion vision.

### Genes Required for Photoreceptor Differentiation and Survival

**Figure 3:**
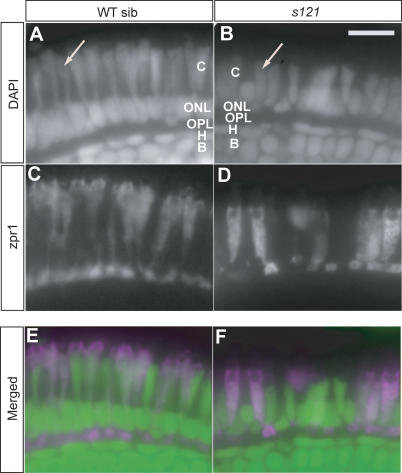
Example of a Mutant with Abnormal Morphology of Cone Photoreceptors Photoreceptors in a retinal section stained with DAPI (A and B) and a marker for double cones, zpr1 (C and D) at 7 dpf in WT larva (A, C, and E) and *yoi^s121^* mutant retina (B, D, and F). Merged images of DAPI (in green) and zpr1 (in magenta) are also shown (E and F). Both zpr1-positive and zpr1-negative cone photoreceptors in the mutant are “stumpy” when compared to those in the control retina (arrows). B, bipolar cells; C, cone photoreceptor cells; H, horizontal cells; ONL, outer nuclear layer; OPL, outer plexiform layer. Scale bar is 10 μm.

**Figure 4:**
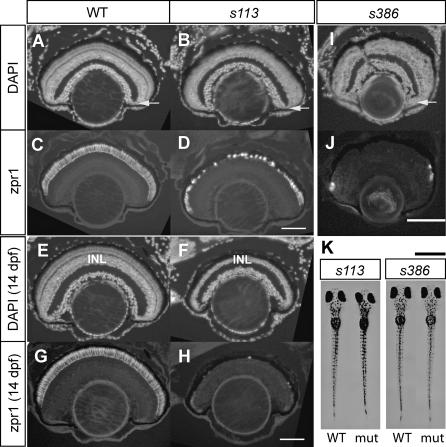
Examples of Mutants with Photoreceptor Degeneration (A–J) WT and mutant retinas (A–H, *mti^s113^*; I and J, *ssd^s386^*) were sectioned and stained with DAPI (A, B, E, F, and I) and zpr1 monoclonal antibody (double-cone photoreceptor marker) (C, D, G, H, and J). At 7 dpf, photoreceptors in the central part of the retina have degenerated in both *mti* (A–D) and *ssd* (I–J). In the *mti* retina at 14 dpf, degeneration has spread to the inner nuclear layer (INL). Arrows show the ciliary marginal zone, from which new cells are continually added to the growing retina. Scale bar is 100 μm. (K) Mutants with photoreceptor degeneration may *(mti^s113^)* or may not *(ssd^s386^)* be dark in VBA assay. Scale bar is 1 mm.

We discovered seven genes essential for photoreceptor differentiation and/or maintenance (Figures 3 and 4; Table 1). No other phenotypes could be discovered in these mutants, and at least four of them are adult viable. In two mutants (five alleles of *wud* and *yoi^s121^*), cone photoreceptors are present, but their shapes are shorter and thicker than in WT (see Figure 3). This “stumpy” morphology is not restricted to one particular cone type, as shown by labeling with zpr1, a double-cone-specific marker (Figure 3C and 3D). In five mutants (five alleles of *mti,* as well as *gosh^s341^, pday^s351^, lim^s382^,* and *ssd^s386^*), all photoreceptors are lost before 6 dpf, except for a small population in the margins of the eye (Figure 4A–4J), where proliferation and differentiation of neuronal precursors continue throughout the life of the fish [23]. This suggests that some of the newborn cells select the photoreceptor fate, but die shortly after beginning differentiation. In *mti* mutants, degeneration spreads to the outer part of the inner nuclear layers (Figure 4F and 4H). This mutant is also the only one in this class with defective VBA (Figure 4K), as examined further below.

Six of the seven photoreceptor-defective mutants appear normal in their VBA response to light (Figure 4K). This is a curious finding, as it may suggest that classical cone/rod-mediated photoreception is not strictly required for this neuroendocrine response. It is conceivable that the pineal gland, a light-sensing organ in the dorsal forebrain, may control the VBA instead of, or together with, the retina. We therefore asked if presence of the VBA correlated with an intact pineal in our photoreceptor-degeneration mutants. Both VBA-normal and VBA-defective mutants showed a normal pineal, based on expression of shared marker zpr1 (Figure S1). This suggests that none of the mutated genes found here are necessary for the maintenance of the pineal photoreceptors. Moreover, it implies that pineal photoreceptors are not sufficient to control the VBA. This is consistent with the observation that *lakritz* mutants, which completely lack all RGCs due to mutation in the atonal homolog *atoh7 (ath5),* but which apparently have a normal pineal gland, show an extreme VBA defect (VBA = 0) [17]. Based on these combined genetic data, we propose that classical cone/rod photoreception is dispensable for this behavior and that other photosensitive cells, situated in the inner retina, signal ambient light levels to the VBA circuitry via the optic nerve.

### Genes Required for General Visual Function, Including Phototransduction and Adaptation to Sudden Increases in Light

**Figure 5:**
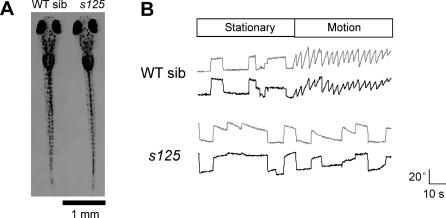
Example of an OKR Mutant with Normal Morphology (A) WT sibling and *zat^s125^* mutants are indistinguishable in their appearance (shown here at 6 dpf). (B) The mutant showed no OKR, but saccadic eye movements, which were not correlated to the motion stimulus. The *zat* gene encodes cone-specific Gc3.

We identified 11 mutant alleles of nine genes *(bld^s394^, dada^s503^, dln^s518^, dln^s393^, edpo^s371^, laj^s304^, mzr^s130^, nof^s377^, snev^s102^, zat^s125^,* and *zat^s376^)* without detectable anatomical defects (unpublished data), but with complete absence of OKR and OMR (both indices 0.1 or less) (Figure 5; Table 1). The *nof^s377^* mutation is a new allele of the alpha subunit of cone transducin [24], and the *zat* gene was shown by positional cloning to encode cone-specific guanylyl cyclase, Gc3 (unpublished data) [25]. Based on these findings, it is likely that some of the other seven genes in this category also encode components of the phototransduction cascade.

**Figure 6:**
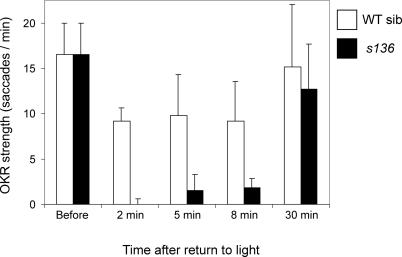
Example of a Mutant with a Potential Defect in Light Adaptation OKR is plotted at several time points before and after dark treatment for 45 min. WT sibling larvae (*n* = 6) recover quickly from the dark pulse, while *nki^s136^* mutants (*n* = 6) show reduced responsiveness for several minutes after return to the light. Average number of saccades to a constant motion stimulus is shown for each time point. Error bars indicate standard deviation.

Other mutants were found to have variable visual impairments. We speculated that some of these mutants were unable to adjust the gain of their visual responses due to defective light adaptation. We therefore rescreened mutants with partial impairments and normal histology, using a behavioral paradigm previously developed by us to test this process in zebrafish larvae [19]. In brief, initially light-adapted fish were placed in a dark environment for a period of 45 min and then tested for OKR after return to light. The recovery of visual responsiveness following the sudden transition from dark to light served as a convenient surrogate measurement for light adaptation, although we do not know how closely this paradigm mimics adaptation. We identified five mutants *(nki^s136^, uta^s301^, utut^s357^, ymj^s392^,* and *mdr^s527^)* in which the measured light adaptation was severely delayed (example in Figure 6). In addition, another mutant, *nbk^s342^,* had a chronic impairment of both OKR and OMR, which varied with genetic background and occasionally improved with repeated stimulus presentation (unpublished data). The mutated genes may be components of light-adaptation pathways, either in photoreceptors or in the retinal network.

### Genes Required for Ganglion Cell Differentiation and Axon Pathfinding

**Figure 7:**
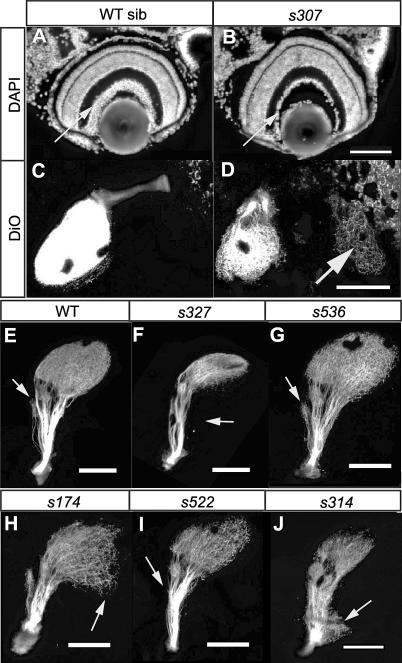
Examples of Retinofugal Projection Mutants (A and B) Sections of WT and *boj^s307^* retina stained with DAPI. The mutant retina has a thinner RGC layer (arrow). (C and D) Dorsal views of RGC axons from the right eye of a WT and a *boj^s307^* mutant labeled with DiO, showing mutant axons in the ipsilateral tectum (arrow). To show that there is no ipsilateral projection in WT, the image is overexposed. (E–J) Lateral views of RGC axons labeled with DiO after removal of the eye. Anterior is to the left, dorsal to the top. In WT, the tectum and other retinorecipient areas are clearly visible (E). The arrow indicates AF-4. In *darl^s327^,* the ventral branch of the optic tract is missing (arrow), and only dorsal tectum is innervated (F). In *walk^s536^,* innervation of AF-4 (arrow) is disorderly (G). In *exa^s174^,* the posterior tectum (arrow) appears to be incompletely innervated, while AF-4 is larger than in WT (H). In *miss^s522^,* AF-4 (arrow) is reduced in size (I). In *mich^s314^,* there is an ectopic arborization (arrow) at the root of the optic tract (J). Scale bars are 100 μm.

In WT animals, RGCs project to the contralateral brain and terminate in ten different arborization fields (AFs), of which AF-10, the tectum, is the largest [26]. In our collection of behavioral mutants, we found eight new mutants with specific retinofugal projection deficits (Figure 7): *boj^s307^, darl^s327^, walk^s536^, exa^s174^, miss^s522^, mich^s314^, drg^s510^,* and *drg^s530^,* as well as a new allele of *bel.* In *bel^s385^* mutants, RGCs develop normally, but project, in variable proportions, to the ipsilateral side of the brain. The new allele was discovered in the OKR screen, because mutants showed reversed eye movements in response to a drifting grating, as is expected from a predominantly ipsilateral projection [9,27]. The reversed response is seen only when the grating rotates around the mutant, as in the OKR assay, because in this situation the direction of motion is opposite between the two eyes (e.g., temporal-to-nasal for the right eye and nasal-to-temporal for the left eye). In the OMR assay, both eyes are exposed to motion flowing in the same direction. Consequently, the OMR of *bel* mutants is intact.

The RGC layer of *boj^s307^* mutants is dramatically reduced to about a third of that in WT (Figure 7A and 7B). The optic nerve is thinner, and a variable fraction (up to 50%) of the remaining RGC axons project ipsilaterally (Figure 7C and 7D). Although the axons make this abnormal choice at the midline, they nevertheless show appropriate targeting on the ipsilateral side, innervating the optic tectum as well as the other major AFs. The *boj* mutation complements mutations in both *lakritz* (encoding Atoh7/Ath5) [17] and *daredevil* (encoding an unknown protein) [28], two previously described genes important for RGC genesis or differentiation. The *boj* mutants are visually impaired to variable degrees, but most severely in the OMR. Based on our finding that the OMR is normal in *bel,* the OMR deficit in *boj* is likely due to the reduced number of RGCs, rather than the ipsilateral projection. Another possible cause could be an as-yet unknown patterning defect in the brain, which is often found in ipsilateral RGC projection mutants [29].

**Figure 8:**
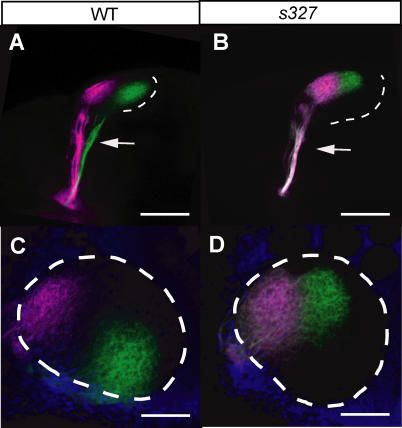
The *darl* Mutant Shows Retinotectal Mapping Deficits (A and B) The nasal-dorsal quadrant of the retina was labeled with DiO (green), and the temporal-ventral quadrant was labeled with DiD (magenta). In *darl^s327^,* the ventral branch of the optic tract is missing (arrow). Scale bar is 100 μm. (C and D) Dorsal view of the tectum in the same larvae as in A and B. The ventral half of the *darl^s327^* tectum is not innervated by the dorsal-nasal RGC axons. Anterior is to the left and ventral is to the bottom. Tectal neuropil is demarcated by the dotted line, based on DAPI counterstaining (blue). Scale bar is 50 μm.

In *darl^s327^* mutants, the ventral branch of the optic tract is completely missing, and with it AF-2, AF-3, and AF-6; the dorsal optic tract (with AF-4, AF-5, AF-7, AF-8, and AF-9) appears intact (Figures 7F and 8). The tectum has normal size and histology, but only its dorsal half is innervated at 7 dpf; the ventral half is devoid of retinal input. We asked if the dorsal RGCs, which project their axons to the ventral branch of the optic tact in WT fish (Figure 8A), are missing in *darl^s327^* mutants. We detected differentiated RGCs throughout the retina, including the dorsal part (Figure S2). Axon tracing, following injection of 3,3′-dioctadecyloxacarbocyanine (DiO) and 1,1′-dioctadecyl-3,3,3′,3′- tetramethylindodicarbocyanine (DiD) into the nasal-dorsal and temporal-ventral quadrants of the eye, respectively, revealed that the dorsally located RGCs project into the dorsal, instead of the ventral, branch of the optic tract, sharing the same route as the ventral RGCs (Figure 8B). The absence of both the ventral optic tract and the ventral innervation of the tectum (Figure 8B and 8D) suggests that the *darl* gene is required for specifying dorsal RGC fate. Positional information along the temporal-nasal axis of the retina seems unaltered in the mutant. Despite the severity of the anatomical defect, this mutant's OMR and OKR scores are not substantially reduced. The VBA, however, is severely disrupted, suggesting that this neuroendocrine behavior requires input from dorsally specified RGCs.

The mutants *walk^s536^, exa^s174^,* and *miss^s522^* show specific axon targeting defects, best seen in, but not restricted to, AF-4. AF-4 is associated with the dorsal branch of the optic tract and normally has a well-ordered, compact structure (see Figure 7E). In *walk^s536^* and *exa^s174^,* AF-4 is overelaborated and located at a greater distance from the optic tract (see Figure 7G and 7H). The tectum in the *exa^s174^* mutant shows an abnormal shape, particularly in the ventral-posterior region (Figure S3), and AF-9 is often missing or reduced (unpublished data). In *miss^s522^* mutants, on the other hand, AF-4 and AF-9 are reduced in size or undetectable (see Figure 7I). This mutant is completely unresponsive to motion, while the *walk^s536^* and *exa^s174^* mutants show residual OKR and OMR (Table 1). In all three mutants, AFs associated with the ventral tract appear normal. This observation, together with the finding that OMR and OKR are intact in *darl^s327^* mutants, which lack the ventral tract, suggest that one or more AFs in the dorsal tract play a key role in OMR and OKR.

In *mich^s314^* mutants, a subset of RGC axons make an abnormal turn shortly after crossing the midline and stall to form an ectopic AF (see Figure 7J). The location of this new retinorecipient area is highly consistent among individual mutants. Another OMR mutant, *shir^s362^,* has a severely retarded retinofugal projection at 5 dpf, which recovers by 7 dpf, although the dorsal optic tract remains thinner (Figure S4). Finally, in *blin^s573^* mutants, axon arbors in the tectal neuropil are disorganized and, in *drg* (two alleles), a subset of the RGC axons project to the incorrect layer of the tectum [28]. The axon-targeting phenotypes described here are, for the most part, so subtle and localized that they would have escaped previous lipophilic carbocyanine dye-tracing screens [30].

### Genes Apparently Required for the Function of Specific Behavioral Pathways

Two mutants, *ofrt^s373^* and *amj^s391^,* show severe VBA defects with only minor OKR and OMR impairments. Strikingly, the VBA of *amj^s391^* is reversed: The mutant turns dark in the light and light in the dark, which is the opposite of what is seen in WT. At what stage the photoresponse is inverted in this mutant will have to be elucidated. In addition, we discovered several mutants with VBA defects, but normal OMR and OKR, which are not included in Table 1.

Two other VBA mutants, *dpg^s128^* and *jako^s326^,* showed normal OKR, but were impaired in the OMR. This selective deficit could not be explained by a locomotor problem, as both mutants show normal SSA and are adult viable. Specific deficits such as these may be either due to differential sensitivity to the stimuli presented in the two assays or due to differential effects of the mutation on the underlying neural circuits. Thus, our screen has discovered a small number of mutations that dissociate visual pathways underlying OMR and OKR.

### Genes Required for Posture, Swimming, or Eye Movements

While we did not systematically keep OMR mutants with swimming defects or OKR mutants that did not move their eyes, we saved a small number of mutants whose phenotypes appeared to be informative with regard to specific neural pathways. The morphologically normal *beat^s348^, pah^s374^, slak^s564^,* and *flan^s513^* mutants showed reduced OMR and/or OKR in combination with motor abnormalities. The *pah* gene was positionally cloned and shown to encode phenylalanine hydroxylase, an enzyme required for tyrosine and catecholamine synthesis (unpublished data). These mutations appear to primarily affect motor or other nonsensory central nervous system functions, although additional defects in visual processing may also be present.

## Discussion

In this study, we took a forward genetic approach to identify genes involved in zebrafish visually controlled behaviors. In order to capture a large number of mutants, we screened almost 2,000 F2 families and cast a wide, dense net by screening with three complementary behavioral assays. We report here on the initial characterization of 53 specific mutations in 41 genes, only two of which had previously been described.

### OKR versus OMR versus VBA as Screening Assays

Choice of a suitable assay is paramount to the success of any genetic screen. We found that each of the three assays employed had its specific strengths and limitations. The OKR assay requires each fish to be mounted individually, dorsal side up, in methylcellulose and is therefore much more time-consuming than the OMR assay, for which each group of fish can just be poured into an elongated tank. The OKR assay therefore dictated the pace of the screen, and we were thus unable to test as many fish as with the OMR assay (and may therefore have missed some mutants). However, since the OKR assay records fish individually, whereas the OMR assay records a population, the OKR has the potential to find less-penetrant phenotypes than the OMR. In the primary screen, OMR and OKR assays each discovered a largely nonoverlapping set of visual mutants, which, upon retesting, showed defects in either assay. Thus, the high throughput of the OMR assay complemented the specificity of the OKR assay. This tradeoff also applies to genetic linkage mapping, which we have so far completed for 25 of the 41 loci. We found that the OMR is most useful for presorting of mutants, while the OKR is most suitable for the subsequent “weeding-out” of false positives. The VBA response, on the other hand, is extremely effective in sorting mutants for linkage mapping, but is less suited as a primary screening assay, because it is prone to missing important mutant classes. Screening with all three assays increased the likelihood of finding all mutants and often provided independent confirmation of a behavioral phenotype.

### How Many and What Kinds of Genes Control Visual Behavior?

We found that at least one-quarter, and probably more than half, of the behavioral mutations discovered here affect photoreception. Their phenotypes include defects in photoreceptor formation or maintenance, phototransduction, and adaptation to sudden light changes (whose likely cellular and molecular substrate is located in the outer retina). Another sizable fraction (at least a quarter) of mutations affect RGCs and their projections to the brain. As far as we can conclude so far from our ongoing analysis, mutations affecting the development of higher visual centers (beyond the retinofugal projections) are largely absent from our collection. This could mean that the genes involved in the formation of circuits in higher brain regions are either essential for embryonic development (i.e., their loss of function would lead to early lethality), or they are redundant, which would prevent their discovery by classical mutagenesis screens.

The number of genomes screened should have been sufficient to uncover at least one mutation in each gene of interest, based on the mutation rate measured in the F0 founder males. However, the empirical allele frequency clearly contradicts this optimistic scenario. Of the 41 loci in our collection, 35 are represented by a single allele and four by two alleles. The other two genes for which we found five alleles each, *mti* and *wud,* appear to be outliers. Excluding these two loci, and assuming that the probability of finding a mutation follows a Poisson distribution, the number of genes with no hits is estimated at about 150. This back-of-the-envelope calculation shows that our screen was not saturating, and that many more genes may be discovered using our approach. Potential obstacles to future screens include the intrinsic difficulty of detecting mutants in behavior, as opposed to, say, pigmentation (which was used to measure the mutation rate), and the low mutability of some loci, as has been observed in other large-scale zebrafish screens [31,32].

Satisfyingly, we discovered new alleles of several previously identified genes. These include mutants falling within the limits of our screening criteria, such as *bel* and *nof* (Table 1), as well as others with more severe phenotypes, such as *chk* [20], *bru* [21,22], *ome,* and *nok* [21] (unpublished data). It is possible that some of our mutations have generated weak (or maternally rescued) alleles of housekeeping or other essential genes, although the molecular identification of the first set of genes shows that this is not generally the case. For a precise estimate of the number of genes whose mutations lead to specific, nonlethal visual system phenotypes, a much larger screen will have to be carried out.

### Genes Involved in Photopic Vision and Photoresponse Dynamics

Zebrafish fill an important niche for the genetic study of photoreception. Human pattern vision, like that of zebrafish, is largely cone-driven. Because most genetic work has been done on the rod-dominated retinas of rodents, less is known about phototransduction in cones. Here we have already discovered two mutant alleles of *zatoichi (zat^s125^* and *zat^s376^),* the gene for cone-specific guanylyl cyclase (Gc3), as well as a new allele of *nof,* which encodes the alpha subunit of cone transducin [24]. It is likely that there are additional mutants in phototransduction in our collection, and it will be interesting to study their genetic interactions. Zebrafish are appealing for this work, because all their cone opsin genes have been identified [33], and their photoreceptors are amenable for biochemical [34] and psychophysical studies [35].

The visual system operates over a wide range of luminance intensities by adjusting its sensitivity to ambient light levels. At least two adaptation mechanisms are operational in the vertebrate retina, one acting on the phototransduction cascade itself [36–38] and the other on synaptic strengths within the network of neurons [39]. We have discovered five mutants that exhibit delayed recovery of the OKR following a sudden transition from dark to light. These mutants are otherwise normal and adult viable. We speculate that these mutants have defects in light adaptation, although further analyses, such as electroretinogram recordings, will be needed to define and localize the underlying defect. The mutations identified here should provide novel entry points into a molecular dissection of light adaptation.

### Zebrafish Mutants as Human Eye Disease Models

We identified five genes whose mutations result in loss of photoreceptors. Several processes can lead to retinitis pigmentosa or macular degeneration in mammals, including structural defects of outer segments, excessive light illumination, and genetic disruption of the phototransduction cascade, but the molecular mechanisms of cell death induction are largely unknown [40]. Photoreceptors are lost quickly in our zebrafish mutants (over days), in contrast to rodent models of retinal degeneration, in which the same process takes months [40]. This is advantageous for the screening of therapeutic drugs that block photoreceptor degeneration. Tests of pharmacological rescue could be carried out in conjunction with our high-throughput behavioral assays. Our collection of zebrafish mutants with rapidly degenerating cones provides us with novel tools to examine the molecular mechanisms of macular degeneration in a model system that is not only genetically tractable, but amenable to small-molecule screens [41].

### Axon Targeting and Functional Neuroanatomy

Our screen successfully identified a small assortment of specific axon-guidance mutants. These mutants will serve as starting points for the discovery of proteins involved in axon targeting and synaptic specificity in the visual pathway. But their phenotypes are also significant for assigning function to certain pathways in the zebrafish visual system [42]. While most RGCs project to the midbrain tectum, nine smaller areas, or AFs, also receive direct retinal input [26]. Different AFs are innervated by molecularly and spatially distinct subpopulations of RGCs [28] and probably mediate different visual behaviors. Laser ablations have shown that the tectum is required for localization of prey [43], but is dispensable for OMR, OKR, and VBA [44]. An intact AF-7 is also not necessary for OMR or OKR [44]. Some of the new mutants now help us narrow down the optomotor pathway further by providing “lesions” that are impossible to obtain using surgical, pharmacological, or optical ablation techniques. For instance, in the OMR-deficient *miss^s522^* mutant, AF-4 and AF-9 are reduced. This suggests, but does not prove, that one of these underdeveloped AFs is necessary for the OMR. Conversely, *darl^s327^* mutants lack AF-2, AF-3, and AF-6, but have an intact OMR, indicating that these three AFs are dispensable for this behavior. Based on these phenotypes, we predict that either AF-4 or AF-9 (or both) are required for the OMR.

### Conclusions

Systematic forward genetic approaches have been applied with great success to many areas of biology in a variety of model species. Mutants are not only starting points for gene discovery; their phenotypes often elucidate underlying biological mechanisms even before molecular identification of the mutated genes (e.g., [45]). Our behavioral screen focusing on the zebrafish visual system has achieved three major goals. First, the mutant phenotypes found here have revealed novel genes, or new functions for known genes, which can be identified by positional cloning. Second, these mutations provide novel tools to study central nervous system development and behavior, to localize functions in the brain and to explore the ways in which neuronal circuits reorganize in response to genetic perturbations. Third, our unbiased screen is yielding fundamental insight into the genetic architecture of brain functions and their pathologies. A mutational approach to circuit formation and function, while being an essential first step, should be complemented in the future by targeted manipulations of cells and synapses. Zebrafish are slated to become an excellent system for an integrated genetic approach to unravel cellular and molecular mechanisms of behavior.

## Materials and Methods

### Fish strains, mutagenesis, and screening.

We used fish from the TL strain for mutagenesis and crossed them to fish from the WIK strain for linkage mapping (see below). Embryos and larval fish were kept in E3 solution (egg water): 5 mM NaCl, 0.17 mM KCl, 0.33 mM CaCl_2_, and 0.33 mM MgSO_4_ supplemented with 1:10^7^ w/v methylene blue. Mutations in the zebrafish genome were induced in the spermatogonia of 41 founder males (F0) by three to five treatments with ENU (3 mM for 1 h each, at weekly intervals) and bred to homozygosity over two generations, as previously described [31, 32]. Details of the screen statistics and the specific-locus test used to measure the mutation rate are given in Results.

### Genetic linkage mapping.

We used microsatellite-based linkage mapping methods to locate the mutation in the zebrafish genome [46]. Heterozygous carriers of the mutation (in the TL background) were crossed to the highly polymorphic WIK strain. Carrier pairs were identified from this hybrid progeny and mated repeatedly. Clutches were sorted for mutants and nonmutant siblings using behavioral assays (often a combination of OMR to quickly enrich for mutants, followed by OKR of the enriched population for unambiguous identification of mutants). Bulk-segregant analysis was performed using pooled DNA from siblings and mutants. This method involves PCR with a set of 192 polymorphic simple-sequence repeat markers (oligonucleotide primers targeted to unique sequences flanking dinucleotide repeats of variable length [46]). The markers were selected to cover the entire zebrafish genome (25 linkage groups) at roughly even intervals (K. F.-B., unpublished data). Candidate markers showing co-segregation with the mutant pool were confirmed by PCR of single-fish DNA. Map position was further verified by demonstration of linkage to additional markers located in the presumed chromosomal region.

### Complementation tests.

We completed classical complementation crosses among all mutants with similar phenotypes (Table 1) or with reported mutants with similar phenotypes or similar map position (if available). Heterozygous *nof* carriers were obtained from S. Brockerhoff (University of Washington). Heterozygous *bel* carriers were obtained from C. B. Chien (University of Utah). Complementation tests for *nok* were carried out by S. Horne (UCSF). Complementation tests for *bru* were carried out by J. Malicki (Harvard).

### Assessment of VBA.

Fish were kept on the fluorescent illuminator (950 cd/m^2^) for at least 20 min to light-adapt. The pigmentation of the fish was visually scored in four grades to determine the VBA score, with 1 = normal (WT), 0.7 = slightly dark, 0.3 = intermediate dark, and 0 = strongly dark. In this scoring system, the previously discovered, RGC-deficient *lakritz* mutant scored 0 [17] and served as a reference to calibrate the index. The VBA score for variably dark mutants was estimated by averaging over at least ten individuals.

### Recording of the OMR.

The OMR assay was conducted as described previously [16]. Visual stimuli were displayed on a flat-screen CRT monitor that faced upward. The stimuli, which consisted of moving sinusoidal gratings, were generated in MATLAB (MathWorks, Natick, Massachusetts, United States), using the Psychophysics Toolbox extensions (http://psychtoolbox.org). The gamma function of the CRT was measured using a Minolta LS-100 (Tokyo, Japan) light meter, and corrected using MATLAB. The images of the fish before and after each stimulus were captured by a digital still camera (Nikon CoolPix [Tokyo, Japan]), which was triggered by MATLAB using a set of serial commands. These images were downloaded from the camera offline and analyzed using custom macros in Object-Image (http://simon.bio.uva.nl/object-image.html). Ten to 40 larvae (routinely 25) were placed in custom-built acrylic tanks, or “racetracks,” which allowed the larvae to swim in only two directions. Twelve racetracks were placed side by side on the monitor. After subtracting two consecutive images to remove the background, the position of each fish was determined by using the Analyze Particles function of Object-Image. The average position of the fish in each tank before a stimulus was then subtracted from the average position after 30 s of exposure to a standard motion stimulus. The OMR index of a recessive mutant was calculated for stimuli of 100% and 75% contrast by measuring the average distance swum by the 25% weakest responders in a clutch, divided by the distance swum by the 75% best responders. Each stimulus contrast and stimulus direction were repeated four times and the average OMR score was calculated offline.

### Recording of the OKR.

The OKR assay was conducted as described previously [16]. An animation of sine-wave gratings was projected on the internal wall of a drum (height, 6 cm; inner diameter, 5.6 cm), using an LCD projector (InFocus LP755 [Wilsonville, Oregon, United States]) [44]. To focus the image at close distance, a wide-angle conversion lens (Kenko VC-050Hi [Tokyo, Japan]), a close-up lens (King CU+1 [Tokyo, Japan]), and a neutral density filter (Hoya ND4 [Tokyo, Japan]) were placed in front of the projector. Twelve zebrafish larvae were immobilized in 2.5% methylcellulose in E3 egg water with their dorsal sides up in the inverted lid of a 3.5-cm diameter petri dish and placed into the center of the drum. The fish were imaged using a dissecting microscope (Nikon SMZ-800) and a CCD camera (Cohu MOD8215–1300 [Tokyo, Japan]) to observe horizontal eye movements. Sinewave gratings with a spatial frequency of 20° per cycle moving at 10°/s were used. Image-J (http://www.rsb.info.nih.gov/ij/) was used for both stimulus generation and image analysis. Images were captured via an LG-3 video capture board (Scion; http://www.scioncorp.com) at two frames per second with Scion Java Package 1.0 for Image-J Windows. A custom-programmed Image-J plug-in (A. M., unpublished data) was used to calculate the changes in eye angles. The OKR index of a mutant was defined here as the saccade number per minute divided by the saccade number per minute observed in WT.

### Surrogate light adaptation assay.

The dynamics of OKR in response to sudden changes in illumination was measured as described previously [19]. Fish larvae were put in the dark for 45 min to let them dark-adapt, then subjected to the OKR recording at 2, 8, 15, and 30 min after return to a bright environment (2,400 cd/m^2^ underneath the larvae; 400–600 cd/m^2^ at the internal drum wall, where the visual stimulus was projected).

### Recording of spontaneous swimming activity.

Spontaneous swimming activity was measured as described [16]. Larvae at 7 dpf were tested in groups of six fish in a rectangular compartment (3 cm × 7.5 cm) of a four-well, clear acrylic plate (12.8 cm × 7.7 cm [Nunc, Roskilde, Denmark]). Fish images were captured by a digital camcorder (Sony TRV-9 [San Diego, California, United States]) at a rate of 0.5 Hz for 20 min in Adobe Premiere. Recorded movies were analyzed using Image-J. Each frame was subtracted (pixel by pixel) from the previous frame to extract the fish that moved during the inter-frame interval. Spontaneous activity was quantified by counting the number of moving fish across all frames. The SSA index was calculated by dividing the number of movement episodes seen in mutants by that seen in WT siblings.

### Histology and immunohistochemistry.

Zebrafish larvae were fixed in 4% paraformaldehyde in PBS at 4 °C for 2–16 h, transferred to 30% sucrose in PBS plus 0.02% NaN_3_ for 16 h or more, mounted in O. C. T. Compound (Sakura Finetek USA, Torrance, California, United States), frozen, and sectioned at 10–12 μm. In some cases, after fixation, the sample was dehydrated in an ethanol series followed by xylene, embedded in paraffin, and sectioned at 6 μm. For immunohistochemistry, the section was incubated with primary antibodies, fluorescent dye-conjugated secondary antibodies (Molecular Probes, Eugene, Oregon, United States), counterstained with 4′,6-diamidino-2-phenylindole (DAPI), and mounted with Fluoromount-G (Southern Biotechnology Associates, Birmingham, Alabama, United States).

### Fluorescent axon tracing of the optic tract.

Zebrafish larvae were fixed in 4% paraformaldehyde in half-strength PBS at 4 °C overnight. The fish eye was injected with 1% 1,1′-dioctadecyl-3,3,3′,3′- tetramethylindocarbocyanine (DiI), DiD, or DiO dissolved in chloroform [30]. Fluorescent images were observed with a confocal laser-scanning microscope (BioRad MRC 1024 [Hercules, California, United States] or Zeiss LSM [Oberkochen, Germany]).

## Supporting Information

Figure S1 Pineal Photoreceptors Are Present in Retinal Photoreceptor Degeneration Mutants Coronal sections of the forebrain at 7 dpf were stained with DAPI (A, C, E, and G) and zpr1, a marker of both retinal and pineal photoreceptors (B, D, F, and H). Pineal photoreceptors (arrow and inset) were consistently present in mutants in which retinal photoreceptors were depleted (D, F, and H). Scale bar is 100 μm for A–J and 25 μm for the insets.
(1.2 MB PDF)

Figure S2 Dorsal RGCs Are Present and Properly Differentiated in *darl* Mutants Sagittal sections of WT (A and C) and *darl^s327^* retina (B and D) were stained with DAPI (A and B) and zn5 (C and D), a marker for differentiated RGCs. RGCs are present in the dorsal part of the retina and sending out axons into the optic nerve head in the mutant. The mutant eyes are reduced in size compared to WT.
(513 KB PDF)

Figure S3 The Tectum of *exa* Mutants Has an Abnormal Shape RGC axon tracing, following whole-eye DiI fills at 7 dpf, reveals a subtle extension of the tectal neuropil (delineated by DAPI counterstaining) at the ventral-posterior margin (arrow). Scale bar is 50 μm
(1.7 MB PDF)

Figure S4 Retinal Axon Outgrowth Is Delayed in *shir* Mutants Lateral views of the retinal ganglion cell axons labeled with DiO. Anterior to the left, dorsal to the top.
(A and B) At 7 dpf, the retinofugal projection in *shir^s362^* (B) appears similar to WT (A), although the anterior portion may be less dense (arrow).
(C and D) At 5 dpf, RGC axon outgrowth in *shir^s362^* (D) evidently lags behind WT (C). Scale bar is 100 μm.
(1.6 MB PDF)

Video S1 Optomotor Response The movie shows a close-up of part of a racetrack tank during OMR testing. A visible light filter has been used to remove the stimulus, and the fish are visualized using infrared light (Sony TRV-9 video camera, night vision mode). The stimulus is represented below. Initially, a converging grating brings the fish into the field of view. After 8 s, the stimulus changes to a rightward-moving grating, and all the fish swim to the right, out of the field of view. At 18 s, the converging movie reappears, and the fish return. Playback in Quicktime runs at twice the actual speed.
(2.3 MB WMV)

Video S2 Optokinetic Response The WT larva is on the left, and a *zat^s125^* mutant is on the right. For the first 60 s no stimulus is shown, and both fish show spontaneous eye movements. After 60 s, a clockwise-rotating striped pattern is projected on the drum around the fish. The WT fish responds by tracking the pattern slowly to the right and making fast reset saccades to the left. The mutant continues to make undirected spontaneous eye movements.
(2.21 MB MOV)

### Accession Numbers

The GenBank (http://www.ncbi.nlm.nih.gov/) accession numbers of the *Danio rerio* genes discussed in this paper are retinal *guanylyl cyclase 3 (gc3)* (AY050505) and *phenylalanine hydroxylase (pah)* (BC056537).

## Abbreviations

AF: arborization field
[number] dpf: day [number] postfertilization
DiD: 1,1′-dioctadecyl-3,3,3′,3′- tetramethylindodicarbocyanine
DiI: 1,1′-dioctadecyl-3,3,3′,3′-tetramethylindocarbocyanine
DiO: 3,3′-dioctadecyloxacarbocyanine
ENU: ethylnitrosourea
OKR: optokinetic response
OMR: optomotor response
PhR: photoreceptor cell
RGC: retinal ganglion cell
SSA: spontaneous swimming activity
VBA: visually mediated background adaptation
WT: wild-type
