# Supplementary material for: Forward Genetic Analysis of Visual Behavior in Zebrafish
Source: PLoS Genet. 2005 Nov 25;1(5):e66. doi: 10.1371/journal.pgen.0010066 (PMC1287954; doi:10.1371/journal.pgen.0010066)
Supplement: Supplementary file 3 [file pgen.0010066.sg003.pdf]

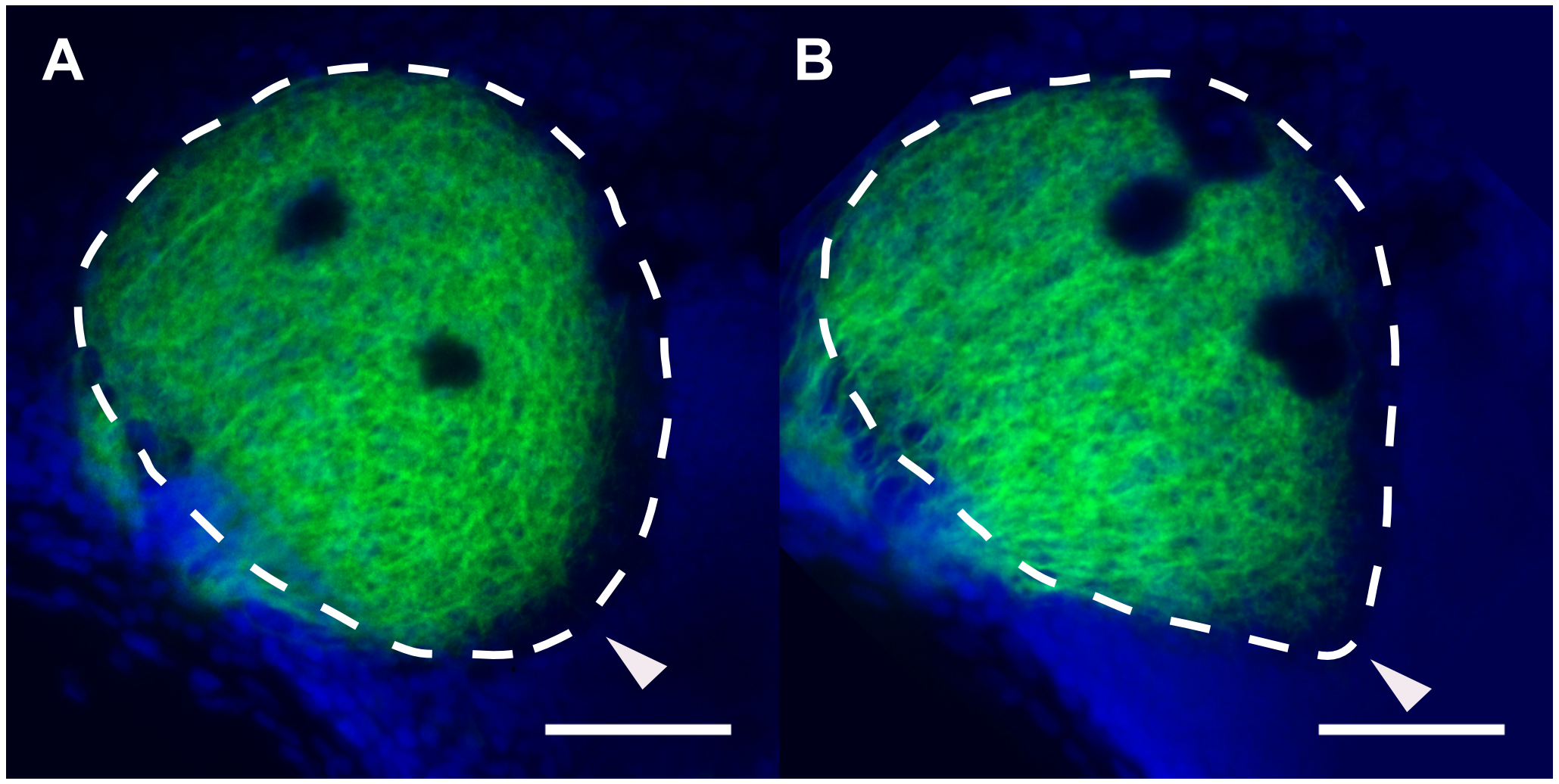

Muto et al., Fig.S3
